# A 15-Day Grazing–15-Day Rest Regime Promotes Plant Diversity and Leaf-Trait Responses in an Alpine Shrub Meadow of the Qilian Mountains, Northeastern Qinghai–Tibet Plateau

**DOI:** 10.3390/plants15121879

**Published:** 2026-06-17

**Authors:** Haijie Zhao, Shaochong Wei, Liang Mao, Qiang Li, Xiaojun Yu

**Affiliations:** 1 Pratacultural College, Gansu Agricultural University, Lanzhou 730070, China; 107332202050@st.gsau.edu.cn (H.Z.); weisc@st.gsau.edu.cn (S.W.); 1073323010027@st.gsau.edu.cn (L.M.); 1073324020055@st.gsau.edu.cn (Q.L.); 2 Key Laboratory of Forage Germplasm Innovation and New Variety Breeding of Ministry of Agriculture and Rural Affairs (Co-Sponsored by Ministry and Province), Lanzhou 730070, China

**Keywords:** alpine shrub meadow, rotational grazing, leaf functional traits, phenotypic plasticity, species diversity

## Abstract

Alpine shrub meadows on the Qinghai–Tibet Plateau are key warm-season pastures that support pastoral production and ecosystem stability in fragile high-elevation regions. Due to low temperatures, short growing seasons, and slow vegetation recovery, these pastures are highly sensitive to inappropriate grazing management. However, the effects of different grazing–rest time configurations on plant community composition and leaf functional traits in alpine shrub meadows remain insufficiently understood. In this study, we evaluated five grazing treatments in an alpine shrub meadow in Sunan County, central–eastern Qilian Mountains: 10 days grazing–20 days rest (T1), 15 days grazing–15 days rest (T2), 20 days grazing–10 days rest (T3), continuous grazing (CG), and grazing exclusion (CK). In the third year of treatment implementation, we measured the community diversity, species importance values, and leaf functional traits of four dominant species: *Elymus nutans*, *Carex tibetikobresia*, *Oxytropis kansuensis*, and *Bistorta vivipara*. T1 and T2 significantly increased species richness, Shannon–Wiener diversity, and Simpson diversity compared with CG and CK. NMDS and PERMANOVA further showed significant differences in overall community composition among grazing treatments. Grazing generally reduced the leaf length, leaf width, and leaf area, whereas T2 showed relatively stronger leaf recovery among grazing treatments. Specific leaf area, specific leaf weight, and leaf length–width ratio showed higher variability and calculated plasticity than leaf thickness and leaf dry matter content, suggesting that resource-acquisition and morphological traits were more responsive to grazing than conservative structural traits. The coefficient of variation of leaf traits was positively associated with the plasticity index, although this association should be interpreted cautiously because both indices were calculated from the same underlying trait dataset. Overall, under the conditions of this three-year, single-site experiment and a target moderate grazing intensity, the 15-day grazing–15-day rest regime performed best among the tested treatments. This regime may provide a practical reference for rotational grazing management in similar warm-season alpine shrub meadows, but its broader applicability requires further validation across different grassland types, grazing intensities, climatic conditions, and longer monitoring periods.

## 1. Introduction

Grassland ecosystems are a major component of the global terrestrial biosphere and play fundamental roles in maintaining regional ecological balance, conserving biodiversity, and supporting livestock production [1,2]. Alpine meadows are widely distributed across the Qinghai–Tibet Plateau and the Qilian Mountains, among which alpine shrub meadows represent important warm-season pastures on the Qinghai–Tibet Plateau [3]. Because these ecosystems are characterized by low temperature, a short growing season, and limited water availability, their vegetation composition, community structure, and ecological processes are highly sensitive to external disturbances, particularly grazing [4]. Human activities have increasingly become key drivers of plant community composition and ecological functioning in alpine meadows [5]. Long-term inappropriate grazing has resulted in vegetation degradation, simplified community structure, and reduced species diversity in some alpine meadows, thereby threatening regional ecological security and the sustainable use of grassland resources.

Grazing is one of the most important grassland management practices [6], and both grazing regime and grazing intensity strongly influence vegetation recovery, species coexistence, and the maintenance of ecosystem functions. Moderate grazing can enhance community diversity by reducing the competitive dominance of tall or highly competitive species and promoting resource redistribution [7]. However, continuous grazing or inappropriate grazing–rest configurations may result in vegetation dwarfing, the loss of palatable forage species, simplified community structure, and grassland degradation. Therefore, compared with controlling grazing intensity alone, optimizing grazing systems, particularly the temporal arrangement of grazing and rest periods, is increasingly recognized as a key pathway for achieving sustainable grassland management [8,9]. This is especially important in alpine meadows, where short growing seasons and slow vegetation recovery make plant communities highly dependent on appropriate recovery intervals between grazing events.

Rotational grazing provides plants with intermittent recovery opportunities by alternating grazing periods with rest periods. This approach can mitigate the continuous pressure imposed by livestock defoliation and trampling and may maintain or enhance community productivity and diversity [10]. Previous studies have shown that rotational grazing can improve community structure and ecological functioning by periodically reducing grazing pressure and providing recovery windows for plant regeneration [11]. Nevertheless, the mechanisms by which different rotational cycles balance disturbance and recovery remain insufficiently understood. This knowledge gap is especially important in alpine meadows, where the short growing season, harsh environment, and slow vegetation recovery may cause distinct community responses to different grazing–rest combinations. Existing research has focused mainly on comparisons between continuous grazing and grazing exclusion or on grazing intensity gradients, whereas systematic studies on different combinations of grazing days and rest days are still limited. In particular, the coordinated responses of community species diversity and plant leaf functional traits to different rotational grazing schedules remain poorly quantified, limiting the mechanistic understanding of grazing-system optimization in alpine meadows.

Plant functional traits provide a crucial link between individual plant adaptive strategies and ecosystem functioning [12]. These traits describe plant morphological, physiological, and ecological attributes under specific environmental conditions and are widely used to predict community assembly, succession dynamics, and ecosystem functional responses [13,14]. Functional traits are commonly expressed through organ-level characteristics, including leaves, roots, and seeds [15]. Among these organs, leaves are particularly important because they are directly involved in photosynthesis, transpiration, and gas exchange, and their morphological and physiological traits are highly sensitive to environmental disturbance [16]. Therefore, leaf functional traits can effectively reflect plant responses and adaptive strategies under grazing disturbance. Grazing can alter plant functional traits by changing resource availability, canopy structure, and selective pressure on plant species. Guo et al. [17] reported that overgrazing-induced grassland degradation can reduce interspecific trait variation while increasing intraspecific trait variation, thereby affecting ecosystem functioning. To adapt to grazing disturbance, plants may adjust their morphological and physiological characteristics across different grazing regimes [18]. In addition to interspecific trait differences, intraspecific trait variation and phenotypic plasticity are increasingly recognized as important mechanisms underlying plant adaptation to environmental change. Phenotypic plasticity refers to the capacity of plants to modify their morphological or physiological traits under different environmental conditions to maintain survival and growth, and it is considered an important strategy for coping with grazing disturbance [19]. Previous studies have shown that resource-acquisition traits generally exhibit higher variability and plasticity, whereas structural or defensive traits tend to be more conservative and stable [20]. However, several key uncertainties remain. First, although plant functional traits have been widely used to explain grassland responses to grazing, most studies have focused on grazing intensity or simple comparisons among grazing types, while less attention has been paid to how different rotational grazing cycles, especially different grazing–rest period configurations, affect leaf functional traits and their plastic responses. Second, the coordinated responses of plant community diversity, leaf functional traits, and phenotypic plasticity under rotational grazing remain insufficiently understood. Third, the relationship between leaf trait variability, commonly expressed as the coefficient of variation, and phenotypic plasticity, expressed as the plasticity index, has rarely been quantified under grazing disturbance. Clarifying these relationships is essential for determining whether trait variation represents random fluctuation or an adaptive response, and for understanding how alpine meadow plants balance resource acquisition and structural conservation under the combined pressures of harsh alpine environments and grazing disturbance.

Based on the research background and scientific questions outlined above, we proposed the following hypotheses: (1) a rotational grazing regime with 15 days of grazing followed by 15 days of rest would optimize the balance between grazing disturbance and vegetation recovery, thereby increasing plant species diversity in alpine shrub meadows; (2) grazing disturbance would drive shifts in leaf functional traits along the resource acquisition–conservation spectrum, with acquisition-related traits exhibiting greater variability and phenotypic plasticity; and (3) the coefficient of variation (CV) of leaf functional traits would be positively correlated with the phenotypic plasticity index (PI), indicating that trait variability and plasticity may jointly contribute to plant adaptation to grazing disturbance. To test these hypotheses, we conducted a field experiment in an alpine shrub meadow in Sunan County, on the northeastern margin of the Qinghai–Tibet Plateau. Five grazing treatments were established: 10 days of grazing followed by 20 days of rest, 15 days of grazing followed by 15 days of rest, 20 days of grazing followed by 10 days of rest, continuous grazing, and grazing exclusion. We then examined the dynamics of plant species diversity and leaf functional traits of four dominant plant species under different grazing regimes.

## 2. Materials and Methods

### 2.1. Study Area

The field experiment was conducted in Huangcheng Town, Sunan Yugur Autonomous County, Zhangye City, Gansu Province, China (101°50′48″ E, 37°54′35″ N), on the northeastern margin of the Qinghai–Tibet Plateau. The study site is located at an elevation of approximately 3100 m above sea level and has a typical alpine semi-arid climate. The mean annual air temperature ranges from 0 to 1 °C, with mean temperatures of approximately −12 °C in January and 13 °C in July. The growing season lasts approximately 120 days, and the frost-free period is about 95 days. Mean annual precipitation is approximately 260 mm. The vegetation is classified as alpine shrub meadow, which is an important warm-season pasture type in the region. The dominant shrub species are *Dasiphora fruticosa* and *Spiraea salicifolia*. Common herbaceous species include *Elymus nutans*, *Poa pratensis*, *Carex tibetikobresia*, *Potentilla anserina*, *Bistorta vivipara*, and *Polygonum macrophyllum*.

### 2.2. Experimental Design

The field experimental platform was established in 2023 in a warm-season pasture with relatively uniform initial vegetation conditions. Grazing intensity was designed as a moderate level, with a target herbage utilization rate of 55–65%, based on local grazing-management practice for alpine shrub meadows.

From 2023 to 2025, four grazing treatments and one control were established: 10 days of grazing followed by 20 days of rest (T1), 15 days of grazing followed by 15 days of rest (T2), 20 days of grazing followed by 10 days of rest (T3), continuous grazing (CG), and fenced grazing exclusion as the control (CK) (Table 1). The experiment followed a randomized complete block design, with three replicates per treatment. Grazing began on 20 June and ended on 18 September each year, with three rotational cycles arranged during each grazing season. Each grazed plot was stocked with three Tibetan sheep. During the rest periods, livestock were moved to a nearby reserve pasture with baseline conditions similar to those of the experimental plots. The sheep were allowed to graze freely, received no supplementary feed, and were supplied with fresh water daily. To minimize variation in intake and metabolism, two-year-old female Tibetan sheep with an initial body weight of 30.0 ± 2.0 kg were selected. All sheep were vaccinated and dewormed before the experiment.

To ensure that differences among treatments mainly reflected grazing–rest temporal configurations rather than large differences in cumulative seasonal grazing pressure, plot areas were adjusted according to the total number of grazing days in each treatment. Seasonal grazing pressure was calculated as follows: Seasonal grazing pressure = number of sheep × total grazing days/plot area. Based on this calculation, seasonal grazing pressure was approximately 726 sheep-days ha^−1^ for T1, T2, and T3, and approximately 748 sheep-days ha^−1^ for CG. Thus, the grazing treatments were designed to maintain broadly comparable cumulative seasonal grazing pressure while differing in the temporal arrangement of grazing and rest periods. We acknowledge that instantaneous stocking density during active grazing differed among treatments because grazing duration and plot area were jointly adjusted as part of the grazing-regime design.

### 2.3. Sample Collection

Vegetation community characteristics were surveyed in mid-August 2025, during the peak growing season. Leaf functional traits were sampled at the end of each rotational grazing cycle, with sampling conducted once in July, August, and September.

For the vegetation community survey, three representative quadrats (0.5 m × 0.5 m) were established in each plot. All plant species occurring within each quadrat were recorded. Plant cover was measured using the point-intercept method, plant height was determined by measuring the natural height of 10 randomly selected individuals of each species, and frequency was calculated using the quadrat method. After the field survey, aboveground vegetation was clipped at ground level, placed in labeled paper envelopes, oven-dried initially at 105 °C for 30 min, and then dried at 65 °C to constant weight to determine the aboveground biomass.

For leaf sampling, the species with the highest importance value in each major functional group were selected based on the preliminary community survey: *Elymus nutans*, *Carex tibetikobresia*, *Oxytropis kansuensis* and *Bistorta vivipara*. At the end of each grazing stage, 10 healthy individuals of each species were selected from each replicated plot within each treatment. Fifteen fully expanded, intact, and disease-free leaves were collected from each individual by cutting at the leaf base with scissors. Leaf samples were immediately placed in plastic bags and stored in an insulated container to prevent wilting and maintain leaf turgidity. The sampling design was hierarchical. Each grazing treatment included three replicated plots, and the plot was regarded as the experimental unit. Quadrats, individual plants, and leaves were treated as subsamples nested within plots rather than independent experimental replicates. For community variables, the three quadrats within each plot were averaged to obtain one plot-level value. For leaf functional traits, leaf-level measurements were first averaged within each individual and then averaged at the plot level for each species, treatment, and sampling month. These plot-level means were used for subsequent statistical analyses.

### 2.4. Measurement and Calculation of Indices

#### 2.4.1. Community Diversity Indices

Community α-diversity was evaluated using species richness, also known as the Patrick index (*S*), the Shannon–Wiener diversity index (*H*′), the Simpson diversity index (1 − *D*), and the Pielou evenness index (*E*). Species importance value (*IV*) was calculated from the relative height, relative cover, and relative frequency [21]. The formulas were as follows:

(1) IV=(relative height+relative cover+relative frequency)/3

(2) S=total number of species in the quadrat

(3) $$H^{\prime }=-\sum P_{i}{lnP}_{i}$$

(4) $$Simpson\;index(1-D)=1-\sum P_{i}^{2}$$

(5) $$E=H^{\prime }/lnS$$

#### 2.4.2. Leaf Functional Traits

Leaf area (LA, cm^2^) was measured using a leaf area meter (Yaxin-1241; Beijing Yaxinliyi Science and Technology Co., Ltd., Beijing, China). Leaf length (LL, cm) was measured with a straight ruler (DL8203; Deli Group Co., Ltd., Ningbo, China), while leaf thickness (LT, mm) and leaf width (LW, cm) were measured using a Vernier caliper (DL91150; Deli Group Co., Ltd., Ningbo, China) with a precision of 0.01 mm. Leaf fresh weight (LFW, g) was measured using an electronic balance (FA2004; Shanghai Precision & Scientific Instrument Co., Ltd., Shanghai, China). Leaves were then oven-dried at 65 °C to constant weight in an electric thermostatic drying oven (DHG-9070A; Shanghai Yiheng Scientific Instrument Co., Ltd., Shanghai, China), and leaf dry weight (LDW, g) was measured using the same electronic balance. The leaf length: width ratio (LWR), specific leaf area (SLA, cm^2^ g^−1^), leaf dry matter content (LDMC, g g^−1^), and specific leaf weight (SLW, g cm^−2^) were then calculated [22]. The coefficient of variation (CV) and plasticity index (PI) were used to quantify leaf trait variation and phenotypic plasticity, respectively. The formulas were as follows:

(6) LWR=leaf length/leaf width

(7) SLA=LA/LDW

(8) LDMC=LDW/LFW

(9) SLW=LDW/LA

(10) CV=standard deviation/mean×100%

(11) PI=(MAX−MIN)/MAX

where MAX and MIN represent the maximum and minimum mean values of each leaf functional trait within the comparison range for a given species, respectively. To reduce the influence of individual extreme observations, PI was calculated using plot-level mean values rather than raw leaf-level measurements. This index was used as a descriptive, dimensionless measure of relative phenotypic response, allowing for comparisons among traits with different units.

### 2.5. Statistical Analysis

Data processing and preliminary calculations were performed using Microsoft Excel 2016 (Microsoft Corporation, Redmond, WA, USA). Most statistical analyses were conducted using IBM SPSS Statistics 27.0 (IBM Corp., Armonk, NY, USA), while NMDS ordination, PERMANOVA, and the homogeneity of multivariate dispersion test were conducted using the vegan package in (version 2.7.5) in R version 4.5.1 (R Foundation for Statistical Computing, Vienna, Austria) Before analysis, data were tested for normality using the Shapiro–Wilk test and for homogeneity of variance using Levene’s test. The plot was considered the experimental unit in all statistical analyses. Quadrat-level, individual-level, and leaf-level measurements were treated as subsamples within plots and were averaged to obtain plot-level means before statistical testing. Thus, the independent replication for treatment comparisons was based on three replicated plots per treatment. For community diversity indices measured during the peak growing season, one-way analysis of variance (ANOVA) was used to test for significant differences among grazing treatments based on plot-level means. When significant treatment effects were detected, Duncan’s multiple range test was used for post hoc comparisons. For leaf functional traits measured repeatedly in July, August, and September, linear mixed-effects models were used to account for temporal dependence among repeated measurements from the same plots. Grazing treatment, sampling month, and their interaction were treated as fixed effects, while plot was included as the repeated subject. Separate models were fitted for each leaf trait and each dominant species. When significant treatment effects or treatment × sampling month interactions were detected, pairwise comparisons among grazing treatments were conducted within each sampling month using estimated marginal means. Lowercase letters shown in the corresponding leaf-trait figures indicate significant differences among grazing treatments within the same sampling month, rather than comparisons across sampling months. Linear regression analysis was used to examine the descriptive relationships between the coefficient of variation (CV) and phenotypic plasticity index (PI) of leaf functional traits. Statistical significance was set at *p* < 0.05. All values were expressed as the mean ± standard error based on plot-level means.

## 3. Results

### 3.1. Effects of Different Grazing Treatments on Plant Community Diversity and Composition

**Figure 1:**
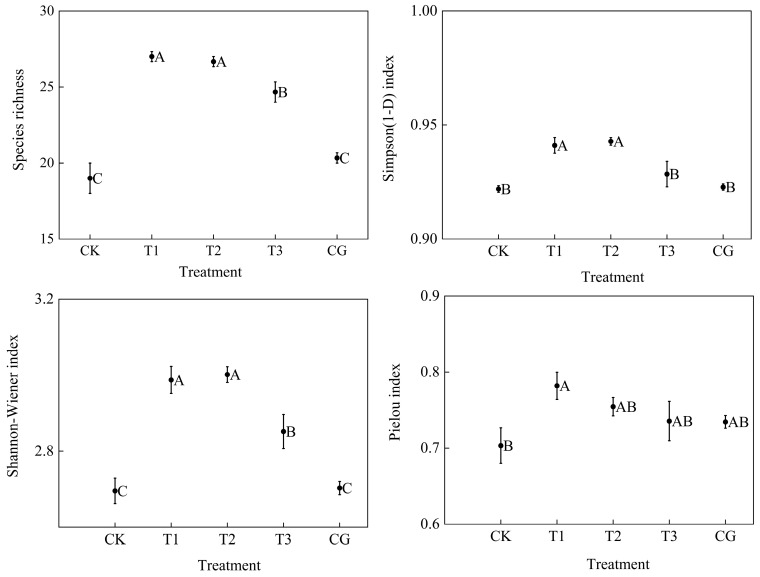
Changes in plant species diversity under different grazing treatments. Different uppercase letters indicate significant differences among treatments (*p* < 0.05).

Plant community diversity differed significantly among grazing treatments (Figure 1). Species richness and the Shannon–Wiener index showed a similar trend. Both indices were significantly higher under T1 and T2 than under CK, T3, and CG (*p* < 0.05). The Simpson index (1 − D) was also higher in T1 and T2 than in the other treatments (*p* < 0.05), whereas no significant difference was observed among CK, T3, and CG. The Pielou evenness index reached its highest value under T1 and was significantly higher than that under CK (*p* < 0.05), while the remaining treatments showed no significant differences.

**Figure 2:**
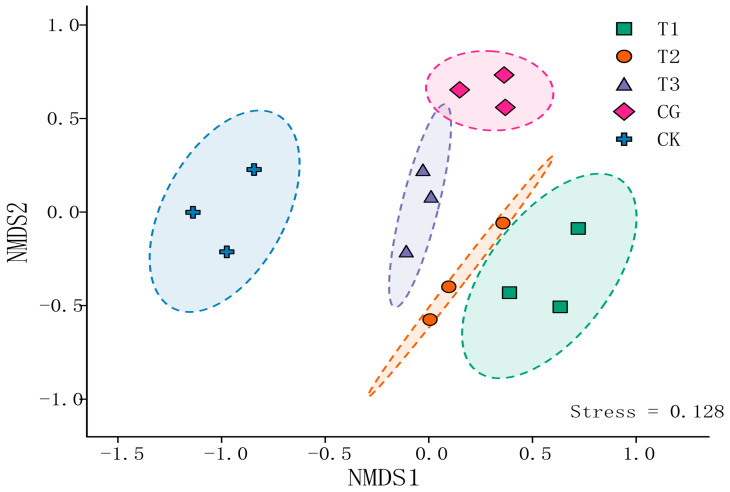
NMDS ordination of plant community composition under different grazing treatments based on plot-level species importance values. Distances among points represent Bray–Curtis dissimilarities in community composition. Different colors and symbols indicate different grazing treatments. Dashed ellipses indicate within-treatment dispersion. The NMDS stress value was 0.128.

Community composition also varied among grazing treatments (Figure 2). The NMDS ordination based on plot-level species importance values showed a clear separation of the treatment groups, with a stress value of 0.128. PERMANOVA confirmed a significant effect of grazing treatment on plant community composition (F = 7.65, R^2^ = 0.754, *p* = 0.001). These results indicate that the differences in species diversity were accompanied by changes in overall community composition. The test for homogeneity of multivariate dispersion was also significant (F = 6.61, *p* = 0.007), suggesting that variation in within-treatment dispersion may have contributed partly to the PERMANOVA result.

### 3.2. Changes in Leaf Functional Traits Under Different Grazing Treatments

**Figure 3:**
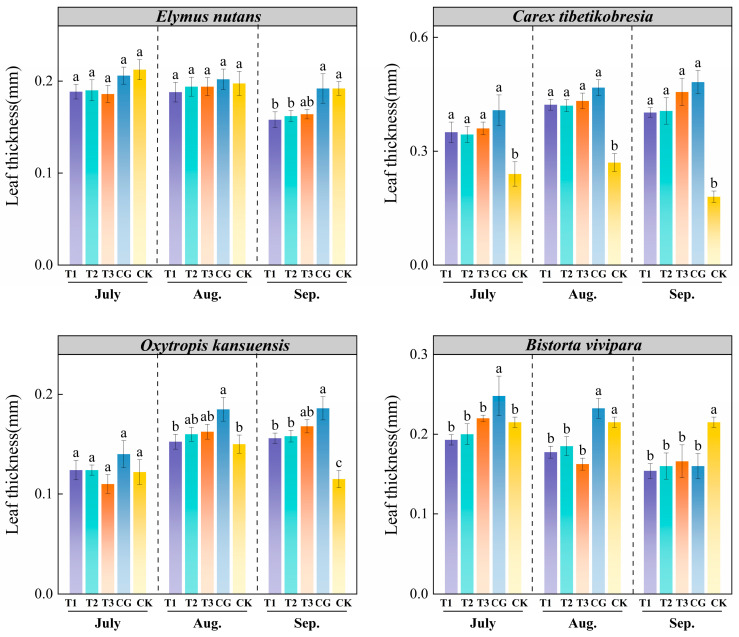
Changes in leaf thickness of the four dominant species under different grazing treatments across the growing season. Values are means ± SE based on plot-level means. Different lowercase letters indicate significant differences among grazing treatments within the same sampling month based on post hoc comparisons following the linear mixed-effects model (*p* < 0.05). n = 3 replicated plots per treatment.

Leaf thickness differed among grazing treatments and showed clear seasonal dynamics (Figure 3). For the grass *E. nutans*, leaf thickness did not differ significantly between grazing treatments and CK during the early (July) and middle (August) growing season, whereas significant differences among treatments emerged in September (*p* < 0.05), with the highest leaf thickness observed under CG and CK. The sedge *C. tibetikobresia* responded more strongly to grazing: throughout the growing season, leaf thickness under all grazing treatments was significantly higher than that under CK (*p* < 0.05), while differences among rotational grazing and continuous grazing treatments were not significant. For the legume *O. kansuensis*, treatment differences in leaf thickness were small in July, but leaf thickness reached the highest values under CG in August and September. For the forb *B. vivipara*, leaf thickness under CG was significantly higher than that under other treatments in July (*p* < 0.05), and leaf thickness under CG and CK was higher than that under the rotational grazing treatments in August (*p* < 0.05).

**Figure 4:**
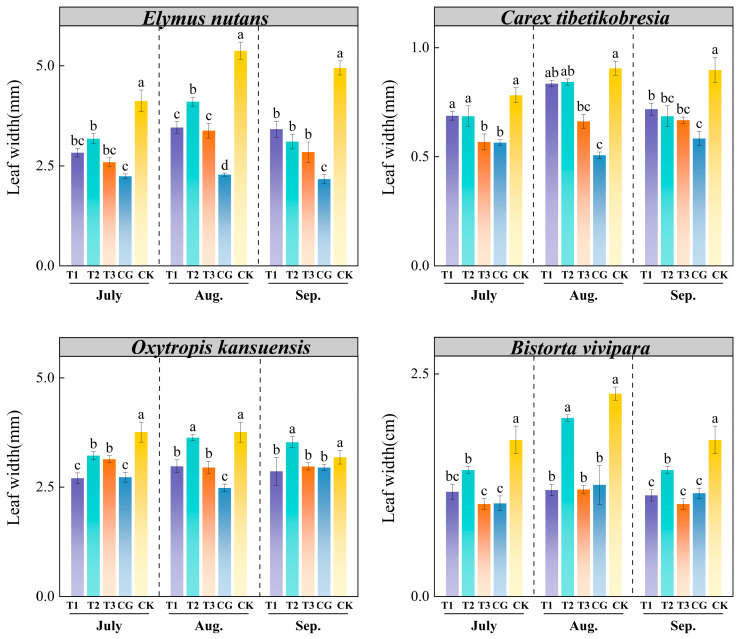
Changes in leaf width of the four dominant species under different grazing treatments across the growing season. Values are means ± SE based on plot-level means. Different lowercase letters indicate significant differences among grazing treatments within the same sampling month based on post hoc comparisons following the linear mixed-effects model (*p* < 0.05). n = 3 replicated plots per treatment.

Leaf width of the four dominant species showed clear seasonal variation under different grazing regimes (Figure 4). Overall, CK generally produced wider leaves, whereas grazing suppressed lateral leaf expansion, especially under CG and T3. For *E. nutans*, leaf width was greatest under CK, and among the grazing treatments, it was relatively higher under T2 and lower under CG. For *C. tibetikobresia*, leaf width under T1 and T2 was significantly greater than that under T3 in July and August (*p* < 0.05). For *O. kansuensis* and *B. vivipara*, leaf width under T2 was significantly greater than that under T1 and T3 during most of the growing season (*p* < 0.05). In particular, in August and September, the leaf width of *O. kansuensis* under T2 did not differ significantly from that under CK, indicating partial recovery of leaf expansion under the 15-day grazing–15-day rest regime.

**Figure 5:**
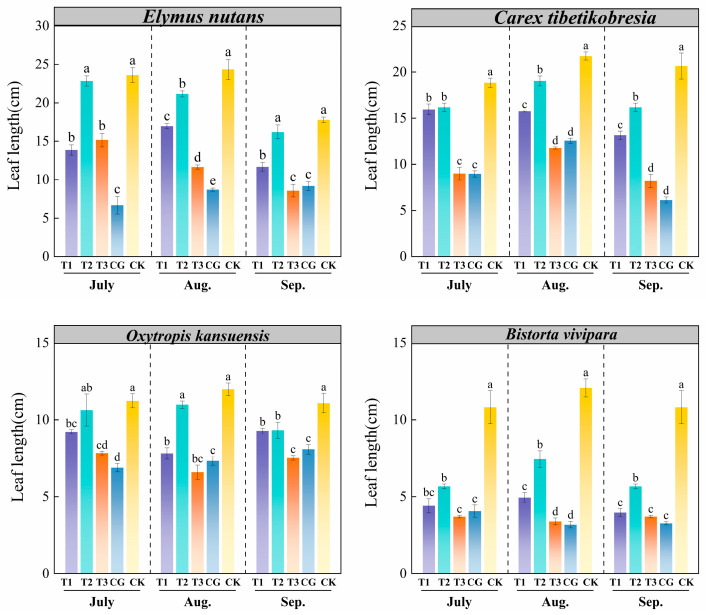
Changes in leaf length of four dominant species under different grazing treatments across the growing season. Values are means ± SE based on plot-level means. Different lowercase letters indicate significant differences among grazing treatments within the same sampling month based on post hoc comparisons following the linear mixed-effects model (*p* < 0.05). n = 3 replicated plots per treatment.

Grazing regime significantly affected leaf length, and responses differed among species (Figure 5). For *C. tibetikobresia*, leaf length under CK was significantly higher than that under all grazing treatments throughout the growing season (*p* < 0.05), while leaf length decreased markedly under CG and T3. Leaf length generally peaked in August, corresponding to the most vigorous growth stage, and values under T2 were generally higher than those under T1 and T3. For *E. nutans*, leaf length under T2 was significantly greater than that under the other grazing treatments (*p* < 0.05) and approached values observed under CK. *O. kansuensis* showed a pattern similar to that of *C. tibetikobresia*, although its sensitivity to grazing disturbance was relatively lower. CK generally maintained longer leaves, whereas CG significantly reduced leaf length. Among the rotational treatments, T2 consistently produced a greater leaf length, particularly in July and August. *B. vivipara* had a lower overall leaf length than the other three species but also responded strongly to grazing regime. Across the three sampling months, CK consistently produced the longest leaves, while among the rotational grazing treatments T2 produced the longest leaves, followed by T1, with T3 and CG showing lower values.

**Figure 6:**
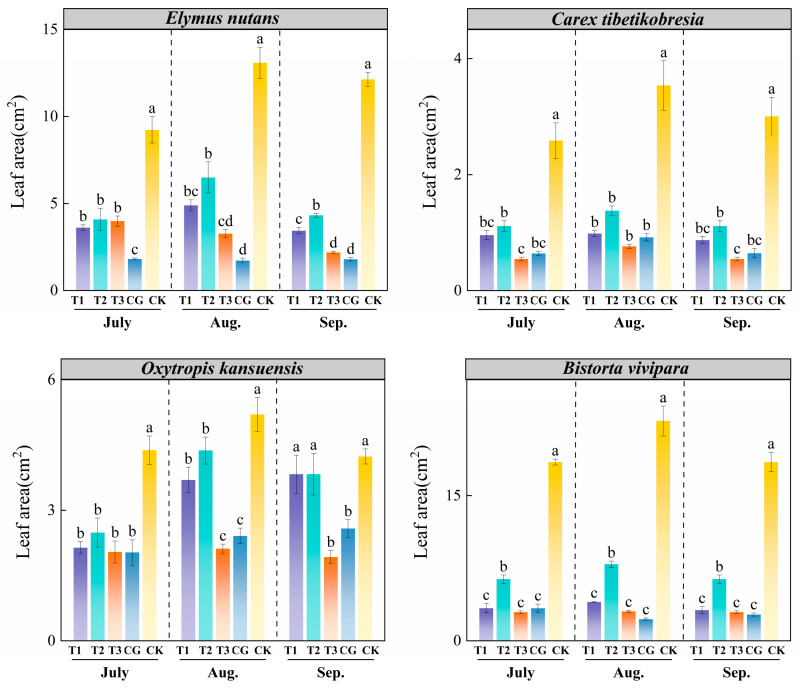
Changes in leaf area of four dominant species under different grazing treatments across the growing season. Values are means ± SE based on plot-level means. Different lowercase letters indicate significant differences among grazing treatments within the same sampling month based on post hoc comparisons following the linear mixed-effects model (*p* < 0.05). n = 3 replicated plots per treatment.

Across the growing season, the four plant species generally showed the greatest leaf area under CK, which was significantly higher than that under grazing treatments, indicating that grazing suppressed leaf expansion (Figure 6). Among the rotational grazing treatments, leaf area was generally larger under T2, whereas it was lower under T3 and CG. Seasonally, the leaf area of all species generally peaked in August and declined in September, indicating a distinct seasonal growth pattern. For *E. nutans* and *C. tibetikobresia*, CK produced the largest leaf area from July to September, T2 maintained relatively large leaf area among grazing treatments, and CG produced the smallest values. For *O. kansuensis*, treatment differences in leaf area were relatively small in July, but leaf area increased markedly under T2 during the middle growing season and approached CK levels. *B. vivipara* showed the clearest treatment differences, with leaf area under CK significantly greater than that under all grazing treatments in each sampling month; among grazing treatments, T2 performed better than T3 and CG.

### 3.3. Coefficient of Variation and Plasticity Index of Leaf Functional Traits

#### 3.3.1. Coefficient of Variation of Leaf Traits

**Figure 7:**
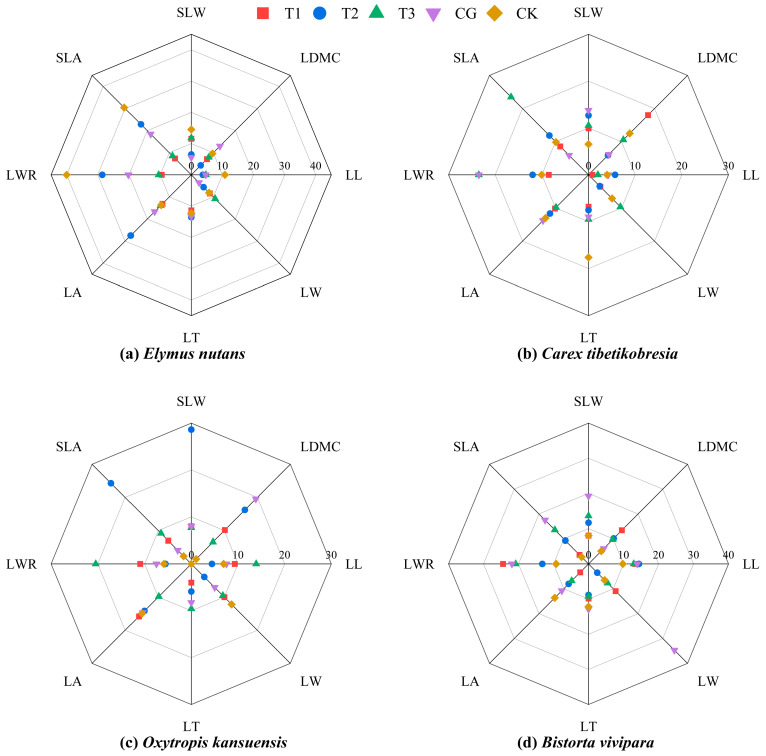
Radar plot showing the coefficient of variation (CV) of leaf functional traits in four dominant species. Larger radial values indicate higher trait variability.

The coefficient of variation of leaf functional traits differed markedly among the four dominant species and among grazing regimes (Figure 7). Overall, the degree of variation differed among traits. SLA, SLW, and LWR showed relatively high variation, whereas LT and LDMC showed lower variation, indicating that structural traits were relatively stable and that resource-acquisition traits were more sensitive to grazing disturbance. In *E. nutans*, leaf-trait variation was mainly concentrated in SLA and LWR, with the highest variation observed under T2, whereas LT and LDMC showed relatively low CV values. *C. tibetikobresia*, *O. kansuensis*, and *B. vivipara* also showed high variation in traits closely associated with resource acquisition, particularly SLW and SLA, and the degree of variation differed among grazing schedules. Overall, the moderate rotational disturbance under T2 appeared to stimulate greater leaf-trait variation, which may help plants adapt to microenvironmental fluctuations and resource redistribution caused by livestock grazing.

#### 3.3.2. Plasticity Index of Leaf Traits

**Figure 8:**
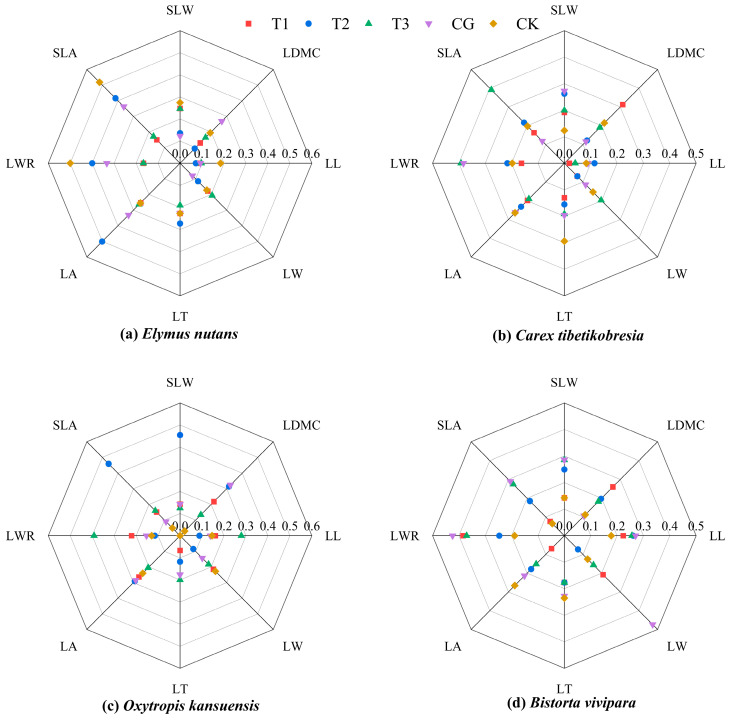
Radar plot showing the phenotypic plasticity index (PI) of leaf functional traits in the four dominant species. Larger radial values indicate higher calculated plasticity.

The plasticity indices of leaf traits showed patterns highly consistent with those of the coefficients of variation (Figure 8). SLA, SLW, and LWR generally had higher PI values, indicating strong phenotypic adjustment capacity of acquisition-related traits under grazing stress. In contrast, LT and LDMC had lower PI values and remained within relatively stable ranges, suggesting that these conservative structural traits were less readily altered by grazing treatments. *E. nutans* showed high plasticity in SLA, LWR, and LA, and T2 produced stronger adjustment across multiple traits. The PI of *C. tibetikobresia* was also concentrated in SLA and LWR, while LDMC showed some plasticity under certain treatments and LT changed only slightly. *O. kansuensis* showed strong plasticity in SLW, SLA, and LWR, especially under T2, indicating strong functional responsiveness under moderate rotational grazing. *B. vivipara* showed higher PI values for LWR, SLA, and LW, suggesting that it adapted to grazing disturbance through adjustments in leaf shape and leaf size. This trait combination, characterized by “conservative structure and flexible resource acquisition”, represents an important strategy by which alpine meadow plants balance growth and defense under complex grazing disturbance.

#### 3.3.3. Descriptive Relationship Between the Coefficient of Variation and Plasticity Index of Leaf Traits

**Figure 9:**
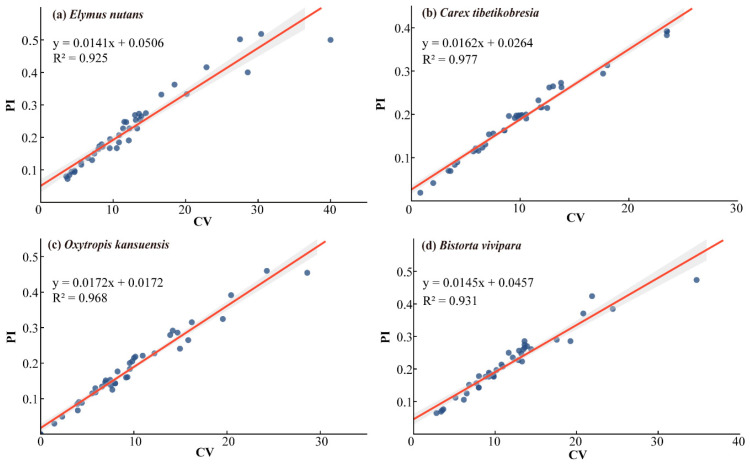
Fitting relationship between the coefficient of variation and plasticity index of plant leaf functional traits.

To further evaluate the relationship between leaf trait variability and phenotypic plasticity under grazing disturbance, linear regression analyses were conducted between the coefficient of variation (CV) and the plasticity index (PI) for the four dominant species (Figure 9). Significant positive relationships between CV and PI were observed in all species, with high explanatory power for *Elymus nutans* (*y* = 0.0141*x* + 0.0506, *R*^2^ = 0.925, *p* < 0.01), *Carex tibetikobresia* (*y* = 0.0162*x* + 0.0264, *R*^2^ = 0.977, *p* < 0.01), *Oxytropis kansuensis* (*y* = 0.0172*x* + 0.0172, *R*^2^ = 0.968, *p* < 0.01), and *Bistorta vivipara* (*y* = 0.0145*x* + 0.0457, *R*^2^ = 0.931, *p* < 0.01). These results show that traits with higher variation tended to have higher calculated plasticity values under the index framework used in this study. However, because CV and PI were derived from the same underlying trait data, this relationship should be interpreted as a descriptive statistical association rather than evidence of a direct causal biological relationship.

## 4. Discussion

### 4.1. Effects of Grazing Regime on Plant Community Species Diversity

Grazing is one of the most important anthropogenic disturbances in grassland ecosystems. By altering interspecific competition and resource distribution, grazing strongly affects community structure and species diversity [23,24]. Its ecological effects depend on the dynamic balance among defoliation pressure, trampling intensity, and vegetation recovery time. In this study, plant community diversity differed significantly among grazing treatments. Species richness, the Shannon–Wiener index, and the Simpson index were all significantly higher under T1 and T2 than under CG and CK. These results indicate that under warm-season alpine shrub meadow conditions in Sunan County, moderate periodic disturbance did not reduce community stability. Instead, it may have helped maintain species coexistence, possibly by reducing the dominance of highly competitive species. This finding is consistent with the intermediate disturbance hypothesis, which proposes that moderate disturbance can weaken the competitive advantage of dominant species, increase niche heterogeneity, and facilitate the coexistence of more species [25].

The community-composition analysis further supported the diversity results. NMDS ordination showed clear separation among grazing treatments, and PERMANOVA confirmed a significant treatment effect on overall community composition. This indicates that the effects of grazing regime were not limited to changes in species richness or diversity indices, but also involved shifts in the relative importance of plant species within the community. The distinct positions of T1 and T2 in the ordination space may be related to the ability of moderate rotational grazing to reduce the dominance of a few highly competitive species while allowing subordinate species to persist. Nevertheless, this interpretation should remain cautious because the homogeneity of the multivariate dispersion test was also significant, indicating that differences in within-treatment variability may have partly contributed to the PERMANOVA result.

Under grazing exclusion, the absence of livestock defoliation and trampling allowed dominant grasses and sedges to occupy the upper canopy and compete strongly for light, thereby suppressing the growth of subordinate forbs in the lower layer. This process can simplify community structure and reduce species richness [26,27,28]. In contrast, under continuous grazing, selective feeding by livestock can reduce palatable species, while frequent trampling can increase soil compaction beyond the tolerance threshold of many plants. As a result, only a small number of grazing-tolerant or chemically defended species may persist, promoting retrogressive succession [29,30,31]. Compared with continuous grazing and grazing exclusion, the T1 and T2 rotational treatments provided appropriate recovery periods for plants, disrupted competitive exclusion, and created opportunities for relatively subordinate species. Periodic grazing reduced the height and cover of dominant species, alleviating their monopolization of light and space, whereas rest periods allowed damaged individuals and suppressed species to recover. This pattern is consistent with the possibility that alternating grazing and rest periods may increase temporal heterogeneity in resource availability and reduce competitive exclusion, thereby maintaining higher community diversity. Nevertheless, niche differentiation and resource redistribution were not directly quantified in this study and therefore require further experimental verification.

Among the rotational treatments, T2 appeared to provide a better balance between grazing disturbance and vegetation recovery. The 15-day grazing period may have reduced the dominance of tall grasses and sedges, while the following 15-day rest period provided time for leaf reconstruction and biomass compensation. In contrast, the longer grazing period and shorter rest period under T3 may have exceeded the short-term recovery capacity of some species. Overall, T2 showed the best performance among the tested treatments in maintaining plant diversity and supporting community composition. The mechanisms underlying these responses remain partly inferential, because soil moisture, soil nutrients, soil compaction, canopy light availability, plant regrowth, and physiological responses were not directly measured in this study.

### 4.2. Response Strategies of Dominant Plant Leaf Functional Traits to Grazing Regime

Leaves are the primary organs responsible for photosynthesis, transpiration, and gas exchange, and their morphological traits are highly sensitive to environmental heterogeneity; therefore, they can serve as effective indicators of ecosystem functional responses [32]. Under grazing disturbance, plants may adjust leaf morphology in response to livestock defoliation and to balance growth requirements with defense or avoidance strategies [33,34,35]. Understanding the relationships between grazing regimes and leaf trait responses can help reveal plant adaptive strategies under disturbance [36]. Li et al. [37] reported that grazing disturbance can reduce the leaf length, leaf width, leaf area, and leaf carbon content. Consistent with this pattern, we found that grazing generally reduced the leaf area, leaf length, and leaf width in the four dominant species. These changes suggest that grazing restricted both longitudinal and lateral leaf expansion, resulting in a smaller leaf area than that observed under CK.

Smaller leaves under grazing may represent a grazing-avoidance or resource-conservation strategy, reducing the probability of defoliation while decreasing water loss and nutrient investment [38]. According to the leaf economics spectrum, plant traits are distributed along a resource acquisition–conservation continuum, with large leaves and high specific leaf area representing rapid resource acquisition, whereas small leaves and high tissue density are generally associated with conservative resource-use strategies [39]. In this study, the reduction in leaf area under grazing suggests a shift toward a more conservative strategy under persistent disturbance. However, leaf length, leaf width, and leaf area under T2 were generally higher than those under T1 and T3, and some values approached those under CK. This pattern indicates that the 15-day rest period may have provided sufficient time for tissue recovery after defoliation. Following moderate tissue loss, plants can compensate by reallocating resources, accelerating new leaf formation, or increasing local growth rates [40].

Seasonal dynamics further supported this interpretation. Leaf length and leaf area generally reached higher values in August, and the T2 treatment showed relatively strong performance during the middle growing season. In alpine meadows, the growing season is short and active plant growth is highly concentrated; therefore, the length of the recovery period after grazing is a key factor determining whether plants can complete leaf reconstruction and biomass compensation. Under T3, leaf recovery was weaker, suggesting that 20 days of grazing caused tissue loss and trampling pressure that exceeded the short-term recovery capacity of plants, while a 10-day rest period was insufficient for complete leaf regeneration. In alpine meadow plants, low temperature and a short growing season constrain photosynthate accumulation and tissue renewal [41]. Once grazing duration becomes excessive, plants may prioritize basic metabolic maintenance and survival over the reconstruction of larger leaves. This response was more pronounced under CG, where continuous grazing pressure restricted leaf expansion and may have promoted a low-input, low-risk organ construction strategy.

Plant adaptation to grazing disturbance was species-specific and closely related to functional group identity. Trait responses to grazing are influenced by plant functional type, species composition, resource-use strategy, and intraspecific variation [42]. In alpine shrub meadows on the Qinghai–Tibet Plateau, plant functional type can help predict the direction and magnitude of species or functional-group responses to grazing gradients [43]. In the present study, the four dominant species did not respond identically to grazing regimes, indicating clear species-specific differences in leaf trait adjustment. Grasses and sedges appeared to be more sensitive to defoliation pressure, with leaf length and leaf area more strongly reflecting grazing intensity. In contrast, legumes and forbs showed more flexible adjustment through leaf thickness, leaf width, or shape-related traits. These differences suggest that community-level responses to grazing were not determined by a single trait or species, but by coordinated and differentiated trait adjustments among functional groups.

Overall, the relatively better performance of T2 at both the community and individual-trait levels suggests that a 15-day grazing–15-day rest regime may provide a more suitable balance between disturbance and recovery in alpine shrub meadows. This regime not only maintained higher species diversity but also allowed dominant plants to retain greater trait flexibility after grazing disturbance. Therefore, T2 may promote a functional balance between resource acquisition and structural conservation, helping plant communities maintain both productivity potential and adaptive capacity under moderate grazing intensity.

### 4.3. Phenotypic Variation and Plasticity Patterns of Leaf Functional Traits

Intraspecific variation in functional traits is an important pathway through which plants respond to environmental heterogeneity, and it plays a key role in community assembly, species coexistence, and ecosystem functioning [44,45]. Exploring intraspecific trait variation can therefore improve our understanding of the adaptive responses of plants to grazing disturbance. Phenotypic plasticity is also a central adaptive mechanism that enables plants to maintain growth and survival across heterogeneous environments [46]. In this study, the coefficient of variation (CV) and phenotypic plasticity index (PI) of leaf functional traits showed highly consistent patterns among the four dominant species. SLA, SLW, and LWR exhibited relatively high variability and plasticity, whereas LDMC and LT remained comparatively stable. These findings indicate that alpine meadow plants do not adjust all leaf traits to the same extent under grazing disturbance. Instead, they appear to preferentially regulate traits closely associated with resource capture, biomass allocation, and leaf morphological adjustment, while maintaining relatively stable structural and conservative traits. This pattern reflects an adaptive strategy characterized by flexible resource acquisition and relatively conservative structural investment.

SLA, SLW, and LWR are closely related to leaf resource-use strategy, biomass allocation per unit leaf area, and leaf shape adjustment [47]. These traits are sensitive to changes in canopy structure, light availability, and resource redistribution caused by grazing-induced defoliation. Therefore, their high variability and plasticity may allow plants to rapidly adjust resource acquisition and organ construction in response to grazing disturbance. In contrast, LDMC and LT mainly reflect the leaf tissue density, structural investment, and physical resistance. For alpine meadow plants experiencing a short growing season, low temperature, and frequent grazing disturbance, large fluctuations in these conservative traits may increase the cost of leaf construction and maintenance, potentially reducing leaf longevity and resistance to damage. Maintaining relatively stable LDMC and LT may therefore be an important strategy for coping with the combined pressures of harsh alpine environments and grazing disturbance [48].

The regression analyses further showed positive relationships between CV and PI in all four dominant species, with high explanatory values (R^2^ > 0.9). These results indicate that traits with greater variation tended to show higher calculated PI values under the index framework used in this study. However, because CV and PI were derived from the same underlying trait data, this relationship should be interpreted as a descriptive statistical association rather than evidence of a direct causal biological relationship. Therefore, the CV–PI relationship should be used only to summarize coordinated patterns of trait variation and calculated plasticity, rather than to infer that trait variability directly drives phenotypic plasticity or adaptive adjustment [49,50]. From the treatment perspective, T2 generally induced higher trait variability and stronger plastic responses, suggesting that moderate periodic disturbance was more conducive to stimulating the regulatory potential of dominant species. Under CK, the relatively stable environment may have provided limited selective pressure for trait adjustment. Under CG or T3, excessive or prolonged grazing disturbance may have forced plants to allocate more resources to basic survival and structural maintenance, thereby limiting flexible trait regulation. Therefore, the advantage of T2 lies not only in maintaining higher community diversity, but also in providing suitable ecological conditions for the expression of phenotypic plasticity in dominant plants.

### 4.4. Limitations and Future Perspectives

This study evaluated the effects of different grazing–rest period configurations on plant community diversity and leaf functional trait responses in an alpine shrub meadow. However, several limitations should be acknowledged. First, this study mainly quantified community diversity indices and leaf morphological traits, while processes such as interspecific competition, niche differentiation, compensatory growth, resource redistribution, soil conditions, canopy light availability, and physiological responses were not directly measured. Therefore, the mechanistic explanations proposed in the Discussion section should be interpreted as plausible ecological interpretations rather than directly demonstrated mechanisms.

Second, the plasticity index used in this study should be interpreted with caution. Although the index is dimensionless and facilitates comparison among traits with different units, it may be sensitive to maximum and minimum values and does not fully describe variation among replicates or complete reaction norms across grazing treatments and sampling months. In addition, because CV and PI were calculated from the same underlying leaf-trait dataset, the observed positive relationship between them should be considered descriptive rather than causal.

Finally, this study was conducted at a single alpine shrub meadow site under a target moderate grazing intensity and was mainly based on observations from the third year after treatment implementation. Although seasonal grazing pressure was calculated and kept broadly comparable among treatments, actual biomass removal, residual biomass, and short-term changes in forage utilization were not continuously measured. Interannual climatic variability may also influence vegetation recovery, grazing tolerance, and leaf-trait responses. Therefore, the applicability and stability of the 15-day grazing–15-day rest regime should be further validated through long-term, multi-site, and multi-gradient experiments.

## 5. Conclusions

Different grazing–rest time configurations significantly affected the plant community diversity, community composition, and leaf functional traits in the alpine shrub meadow studied here. Compared with continuous grazing and grazing exclusion, rotational grazing better maintained community diversity, with T1 and T2 significantly increasing the species richness, the Shannon–Wiener index, and the Simpson index. NMDS and PERMANOVA further showed significant differences in overall community composition among grazing treatments.

Grazing generally reduced leaf length, leaf width, and leaf area, whereas T2 showed relatively stronger leaf recovery among grazing treatments. SLA, SLW, and LWR showed higher variability and calculated plasticity, while LT and LDMC remained relatively stable, suggesting that plants responded to grazing mainly through adjustments in resource-acquisition and morphological traits.

The positive CV–PI relationship indicated that traits with greater variation tended to have higher calculated plasticity values, but this relationship should be interpreted as descriptive rather than causal. Overall, in this three-year, single-site experiment conducted under a target moderate grazing intensity in Sunan County, the 15-day grazing–15-day rest regime performed best among the tested treatments. This regime may provide a practical reference for rotational grazing management in similar warm-season alpine shrub meadows, but its broader applicability should be further tested across different grassland types, grazing intensities, climatic conditions, and longer monitoring periods.

## Figures and Tables

**Table 1:** Experimental design of the grazing treatments.

Treatment	Stage I Grazing	Stage I Rest	Stage II Grazing	Stage II Rest	Stage III Grazing	Stage III Rest	Plot Area (m^2^)	Seasonal Grazing Pressure (Sheep-Days ha^−1^)
T1	10 d (20–29 June)	20 d (30 June–19 July)	10 d (20–29 June)	20 d (30 July–18 August)	10 d (19–28 August)	20 d (29 August–18 September)	1240	726
T2	15 d (20 June–4 July)	15 d (5–19 July)	15 d (20 July–3 August)	15 d (4–18 August)	15 d (19 August–2 September)	15 d (3–18 September)	1860	726
T3	20 d (20 June–9 July)	10 d (10–19 July)	20 d (20 July–8 August)	10 d (9–18 August)	20 d (19 August–7 September)	10 d (8–18 September)	2480	726
CG	20 June–20 July	-	21 July–20 August	-	21 August–18 September	-	3610	748
CK	-	-	-	-	-	-	660	0

## Data Availability

The original contributions presented in the study are included in the article, further inquiries can be directed to the corresponding author.
